# Multimodal Physiological Assessment of Attentional States and Driving Styles in Simulated Driving

**DOI:** 10.3390/s26185883

**Published:** 2026-09-17

**Authors:** Wenchao Zhu, Yingzi Lin

**Affiliations:** Intelligent Human Machine Systems Laboratory, Department of Mechanical and Industrial Engineering, Northeastern University, Boston, MA 02155, USA

**Keywords:** mind wandering, attentional state, multimodal sensing, physiological signals, driver monitoring system

## Abstract

Mind wandering during driving is one of the major sources of road safety risk, but its expression across drivers of different styles remains unclear. Few studies have examined attentional state and driving style concurrently in a 1 h multimodal simulated driving protocol. We collected one hour of driving, five physiological signals, and eye tracking from 48 participants in a simulator. The driving style was obtained from a Multidimensional Driving Style Inventory pre-questionnaire, and attentional state was probed every 120 to 180 s during the experiment. We analyzed feature-level effects with Mann–Whitney U tests, modeled attentional transitions between adjacent attentional states, and compared seven sensor combinations under six classifiers with leave-one-subject-out validation. The study yields three contributions. First, driving style corresponded to distinct multimodal physiological and gaze signatures, and physiology-only features predicted the style label at a balanced accuracy of 0.65, supporting style as a multimodal trait beyond self-report. Second, attention dynamics differed by driving style, with risky drivers drifting from focus to mind wandering twice as often as safe drivers. Third, the optimal sensor configuration depended on the prediction target and driver subgroup: physiology and eye tracking were the most informative sensors for attentional state, while driving style classification depended primarily on physiological features. These results should not be interpreted as evidence for real-time detection. Instead, they provide empirical evidence for sensor selection and suggest that wearable driver-state estimation should be integrated with vehicle- and context-aware monitoring systems.

## 1. Introduction

Driver distraction is one of the major sources of road fatalities, and mind wandering is one of its most challenging factors [1]. Mind wandering refers to off-task internal thought that occurs spontaneously even when a driver intends to focus [2]. Individual differences shape both how often mind wandering occurs and how it affects driving performance [3,4]. These differences emerge from age, personality, and learned driving habits, and they extend to physiological responses during driving [3,4].

A growing literature has analyzed mind wandering during driving and its broad safety implications across studies [5,6]. Mind wandering reduces reaction times to hazards and narrows lane-keeping margins [1,7]. Efforts have also been made to explore objective detection methods of mind wandering using neurophysiological signals such as electroencephalography (EEG), eye tracking, electrodermal activity (EDA), and heart rate variability (HRV) [7,8]. Despite these advances, most driving studies treat the driver as a homogeneous group. Few studies examine how individual traits, such as driving style, shape the behavioral responses of mind wandering [4].

Multimodal sensing has emerged as a primary direction for objective driver-state assessment because physiological, ocular, and vehicle-control signals can capture different aspects of driver states [9,10,11]. Recent benchmark datasets combine EEG, electrocardiography (ECG), electromyography (EMG), EDA, and eye tracking under varied automation levels [12,13]. However, most published studies still rely on one or two modalities, and direct comparisons across sensor configurations remain rare [8,14]. Protocols that capture within-subject state fluctuations alongside between-subject style differences have also rarely been explored in the field. This leaves a gap for richer datasets and systematic sensor comparisons in driver-state assessment.

Understanding individual differences in driving and physiological signals is an essential aspect of developing effective autonomous driving systems. Individual differences refer to the variations in cognitive and executive control abilities among individuals performing the same task [3] and extend to physiological signals as well. Recent research suggests that intentionality and emotional valence are dimensions of individual differences in executive control ability [15]. In addition, individual differences play a significant role in driving behavior and the associated physiological responses [4]. Their performance is influenced by their age, personality traits, driving experience, and habits, such as being more aggressive and willing to take risks, or more cautious and defensive.

### Contributions of the Study

Existing studies have advanced the understanding of driving styles and driver-state assessment, but these topics have largely developed along separate lines. Research on mind wandering or driving styles has mainly examined the behavioral responses of momentary attentional states or driving styles alone, and to our knowledge, no prior study has assessed the contributions of these two factors at the same time. Meanwhile, multimodal studies have demonstrated the feasibility of combining driving, physiological, and eye-tracking signals, but no prior studies have paid attention to whether the temporal transitions of attentional states differ across drivers with distinct driving styles. Therefore, this study aimed to expand the understanding of how driver characteristics related to transitional dynamics of attentional states during prolonged simulated driving.

This study connects these research areas and contributes to the following fields:

(i) It identified a multimodal physiological signature of driving style. Compared to safe drivers, risky drivers showed distinct patterns across driving, physiological, and eye-tracking measures.
(ii) It demonstrated that the transition between attentional states depends on driving styles. Risky drivers showed a lower focus retention rate and more frequent transitions from a focused state to a mind-wandering state.
(iii) It showed that the sensor requirements depended on the prediction target. The optimal sensor for predicting attentional state is the combination of driving parameters and physiological sensors, while predicting driving styles needs physiological sensors as the best set.

This study provides empirical evidence for sensor selection and suggests that wearable driver-state estimation should be integrated with vehicle- and context-aware monitoring systems.

## 2. Materials and Methods

**Figure 1:**
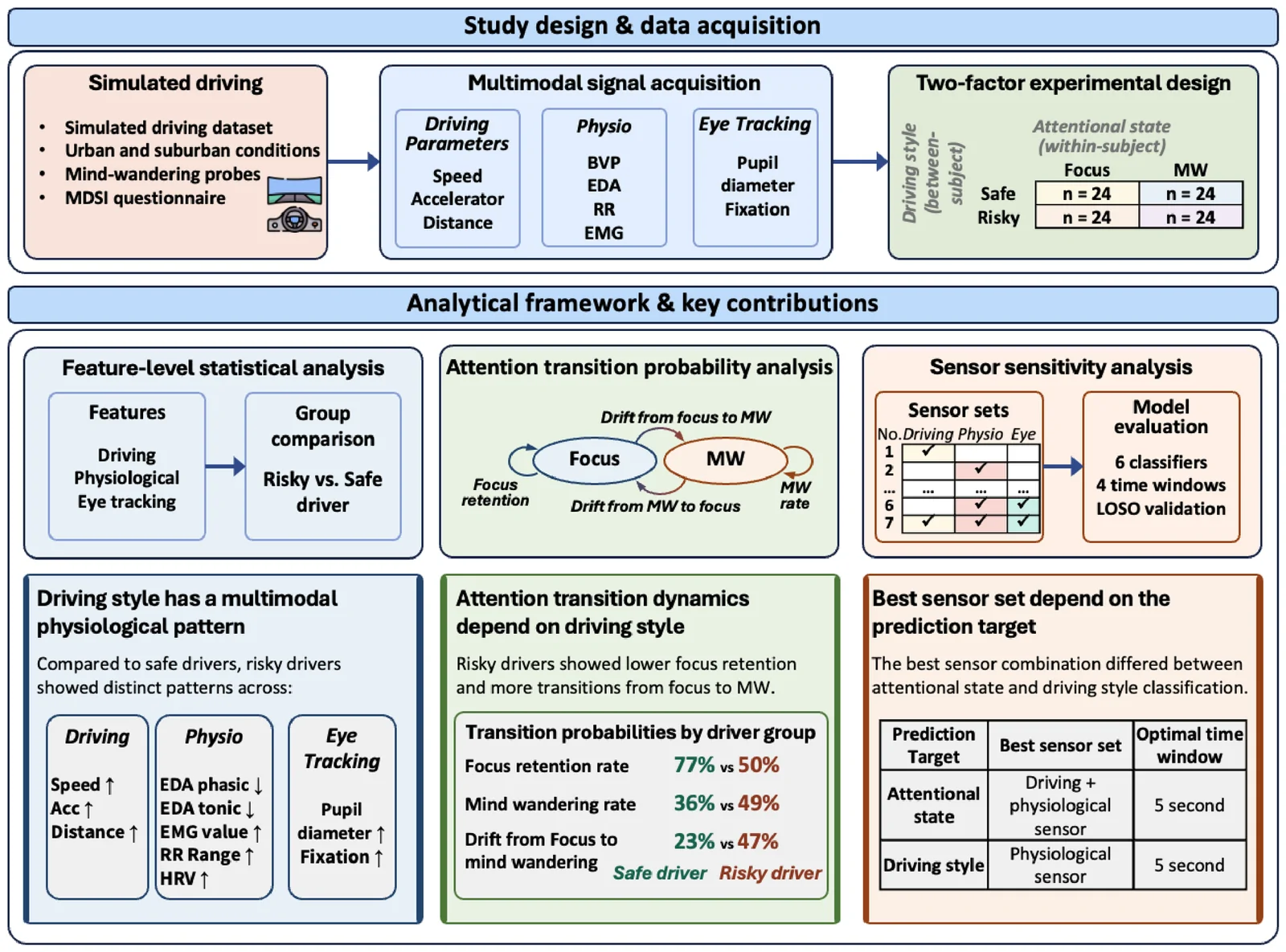
Overview of the multimodal framework for assessing driver attentional state and driving style. The top row presents the study design and data acquisition protocol. The design of experiment was a 2-by-2 factorial design, including driving style and attentional states. The bottom row shows the analytical framework and the key contributions. First, multimodal features were tested for group effects, and driving style was validated to have a distinct physiological pattern. Second, the transition between attentional states were analyzed and the results showed the transition probabilities depends on the driving style. Third, a sensor sensitivity analysis was conducted across sensor sets, time windows, and classifiers to identify the optimal ones. Arrow indicates the analytical flow.Check marks identify the modalities included in a sensor set and ellipses indicate omitted intermediate sensor combination. The matching colors link each analysis to its principal finding.

This study investigates how driver’s attentional states and driving style manifest across driving parameters, physiological, and eye-tracking responses during simulated driving. The overall framework, summarized in Figure 1, is organized in three stages. We first established an experimental protocol that combined a one-hour multimodal driving task with two complementary labels. The first label is a between-subject style label derived from the MDSI questionnaire and the second label is a within-subject attentional state collected via periodic mind-wandering probes. A balanced 2 × 2 experimental design was implemented across 48 participants.

We then implemented an analytical pipeline in which synchronized multimodal signals were preprocessed and segmented into 10 s windows. Features were analyzed both statistically and through a seven sensor-set classification study. This framework is designed to support three contributions that are discussed throughout Section 3: demonstrating correspondence with driving style with multimodalities, characterizing attention as a style-dependent dynamic process, and identifying task- and subgroup-specific sensor configurations. The remainder of this section details the experimental protocol (Section 2.3), data preprocessing (Section 2.4), feature extraction (Section 2.5), and the statistical and classification analyses (Section 2.6).

### 2.1. Participants

The study was approved by the Northeastern University Institutional Review Board (IRB), Boston, MA, USA (protocol code #21-11-09). A total of sixty-seven healthy participants were recruited. The inclusion criteria specified that participants should be healthy and devoid of any medical or psychological conditions that could bias the physiological readings.

### 2.2. Apparatus

Our toolkit for the experiment comprised six sensors to record physiological responses during the simulated driving experience. A Tobii eye-tracker monitored eye movements and pupil dilation as indicators of attention or distraction. The FlexComp Infiniti system from Thought Technology, Montreal, QC, Canada, provided five physiological sensors, including blood volume pulse (BVP), EDA, skin temperature, EMG, and respiratory rate (RR). This set monitored heart rate through a BVP sensor on the non-dominant hand’s middle finger, skin temperature on the back of the same hand, breathing rates via a chest-mounted respiration sensor, skin conductance between the index and ring fingers of the non-dominant hand, and forearm muscle activity through the EMG sensor.

### 2.3. Experimental Procedures

**Figure 2:**
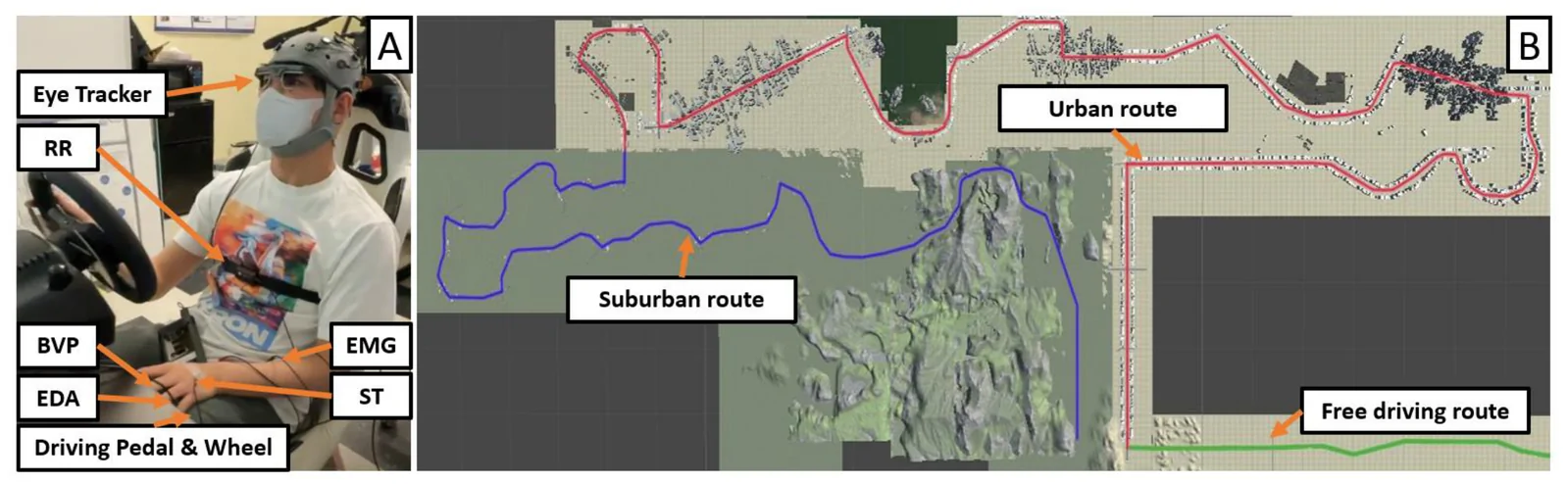
Experiment apparatus. (**A**) Experimental setup and apparatus. (**B**) Road layout of the driving experiment. The green, red, and blue routes represent free driving, urban, and suburban conditions.

The experiment was based on a within-subjects design with three continuous driving routes: free-driving, urban, and suburban conditions (see Figure 2). These scenarios were intended to record participants’ driving performance and physiological responses under different conditions. Evaluation criteria for driving performance were grounded in established traffic laws, focusing on events such as stopping at signs, responding to traffic lights, lane-keeping, and car-following protocols. The urban and suburban driving conditions were created to emulate real-world conditions. The urban setting included commercial zones and residential blocks. The suburban setting contained fewer built-up areas. The virtual reality environment for the experiment was developed using Unity 3D (version 2021.3.21f1) in our lab and replicated real-world driving conditions.

To measure the attentional states during the task, we utilized a mind-wandering probe via a 5-point Likert scale, where 1 point indicated the most mind wandering, or off-task thought, and 5 points indicated the most mind focusing, or on-task thought. Probes appeared every 120 to 180 s. Each probe label was treated as a retrospective report of the immediately preceding attentional state, and features were extracted only from the pre-probe window so that the on-screen question did not contaminate the data. We extracted features from the 5, 10, 15, and 20 s windows before each probe to test the sensitivity of the results to window length.

Before engaging in the simulation, each participant was required to fill out the Multidimensional Driving Style Inventory (MDSI) questionnaire and a mind-wandering questionnaire. MDSI is a scale-based evaluation tool to understand individual driving styles and offers additional context for the recruited participants. Once the participants finished the experiment, they completed the post-questionnaire to assess their experiences and perceptions of mind wandering, its impacts on driving performance, and its potential implications for their on-road driving behaviors.

Terminology was standardized throughout this study. Attentional state refers to the binary within-subject state, including mind-wandering state and focusing state. Mind wandering denotes off-task, internally oriented thought. Focusing denotes on-task attention to driving.

### 2.4. Data Preprocessing

For this study, we synchronized the driving, physiological, and eye-tracking data by resampling them to 50 Hz frequency. For eye tracking, we employed linear interpolation methods to fill in any missing gaps [16]. The BVP signal was filtered via a fifth-order Butterworth band-pass with [0.5, 12] Hz as cut-off frequencies. EDA was filtered via a fifth-order 1 Hz low-pass Butterworth filter. Respiration was filtered via a fifth-order Butterworth band-pass with [0.1, 1] Hz as cut-off frequencies. The first phase of free driving was considered as a practice run and was excluded from the final analysis to minimize any startle effects [17]. Finally, the physiological signals, including BVP, EDA, EMG, temperature, RR, and pupil diameter from eye tracking, were normalized using z-score transformations to ensure unbiased comparative analysis across all participants.

### 2.5. Feature Extraction

Features from the driving parameters, physiological sensors, and eye tracking are listed in Table 1. Literature findings have reported different time windows for analyzing physiological responses, including 2 s [18], 4 s [19], 5 s [17], 7 s [20], 10 s and 30 s [21,22]. In this study, we used the 10 s window as the primary duration to obtain features, and extracted features at 5, 15, and 20 s for the window sensitivity analysis. Our choice of 5, 10, 15, and 20 s pre-probe windows was based on two considerations. First, short-term heart rate variability (HRV) research indicates that RMSSD and SDSD parameters can be estimated reliably from windows as short as approximately 10 s, whereas SDNN requires closer to 30 s for comparable reliability [23]. Our 5 s window therefore sits at the edge of, and our 10 to 20 s windows fall within, the range for short-term HRV features. Second, a fixed 45 s window in some prior driving studies risks either missing brief mind-wandering episodes reported to last as little as 2.5–10 s [24] or may span multiple attentional state transitions within a single window. Therefore, the 5–20 s range was selected to better match the timescale at which short attentional state fluctuations may occur.

### 2.6. Statistical Analysis and Classification Plan

The driving style of each participant was determined using the responses collected from the MDSI questionnaire. This process began by labeling each question as either reflective of ‘safe’ or ‘risky’ behavior. Following this, every response option was assigned a different weighted score for ‘Never’, ‘Rarely’, ‘Sometimes’, ‘Often’, ‘Usually’, and ‘Always’. This process is reversed for questions related to risky behavior. After scoring, the sum of all safety scores was calculated, and the mean was used as a threshold for segmenting the participants into ‘safe’ and ‘risky’ driving styles.

The data were compiled and processed using Python (version 3.11) and the NeuroKit2 (version 0.2.13) package [25]. The analysis plan began with a descriptive analysis of each dependent variable, followed by outlier removal if a value exceeded three times the interquartile range (IQR) above the third quartile or below the first quartile. Next, the Kolmogorov–Smirnov test was employed to check for normality, along with visual validation via histograms and Q-Q plots. Homoscedasticity was tested by Levene’s test. If the data met the assumptions, a two-way ANOVA was conducted to examine the effects of the attentional state and driving style on each feature. Otherwise, the Mann–Whitney U test was used as a nonparametric alternative test to compare these groups for significant differences. An alpha-value of 0.05 was used as a significance threshold. These univariate comparisons are treated as exploratory screening and the interpretation emphasizes effects that are consistent across related features, modalities, and the classifier and feature-importance results.

We classified attentional state and driving style from the extracted features and compared six classifiers: logistic regression, decision tree, k-nearest neighbors, a stochastic gradient descent linear model, AdaBoost, and random forest. Each classifier was tuned by grid search within a five-fold cross-validation and a leave-one-subject-out loop. Balanced accuracy was the primary metric because the attentional state windows are imbalanced; we also report accuracy, macro precision, macro recall, macro F1-score, and confusion matrices. The full pipeline was repeated for the 5, 10, 15, and 20 s pre-probe windows. Seven different feature sets were selected and compared: driving-only, physiological-only, eye tracking-only, driving plus physiological, driving plus eye tracking, physiological plus eye tracking, and all-feature combined.

## 3. Experimental Results

Sixty-seven participants completed the experiments in 2022 and 2023. Four participants were removed from the study due to their failure to complete the experiment, and fifteen participants were removed due to missing eye-tracking or physiological signals. The final sample included 48 participants (22 females and 26 males, mean age 23.2 ± 3.6 years of age).

### 3.1. Driving Style Categorization

**Figure 3:**
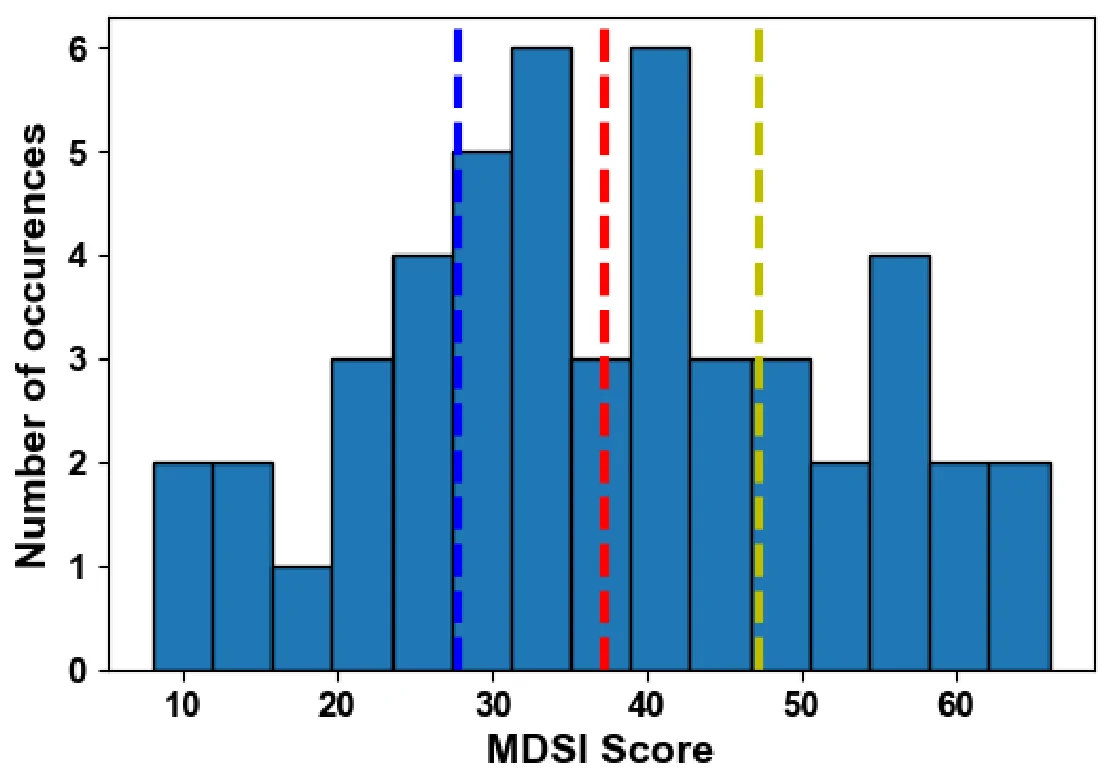
Histogram of MDSI scores. The red line represents the mean score (37.29), the blue line represents Q1 (27.75), and the yellow line represents Q3 (47.25).

The driving style was determined by the safety scores derived from all MDSI questions. Results are shown in Figure 3. These weighted scores were found to follow a normal distribution (Mean = 37.29, SD = 14.64), and the mean served as the threshold to segregate the participants into safe and risky driving style groups. In total, the safe group consisted of 24 participants (11 females and 13 males, mean age 23.6 ± 2.1 years), and the risky group consisted of 24 participants (11 females and 13 males, mean age 22.8 ± 4.7 years).

Driving measures. Four driving parameters were tested for differences between the two driving styles. The speed median was significantly higher in the risky group (Median = 21.629) than in the safe group (Median = 18.205, U = 58,503, z = −3.404, *p* = 0.001). The distance traveled was significantly higher in the risky group (Median = 127.65) than in the safe group (Median = 109.08, U = 58,236, z = −3.436, *p* = 0.001). No significant differences were found between groups for speed standard deviation (*p* = 0.214) or speed range (*p* = 0.195).

### 3.2. Driving Style and Mind Wandering Relationship

**Figure 4:**
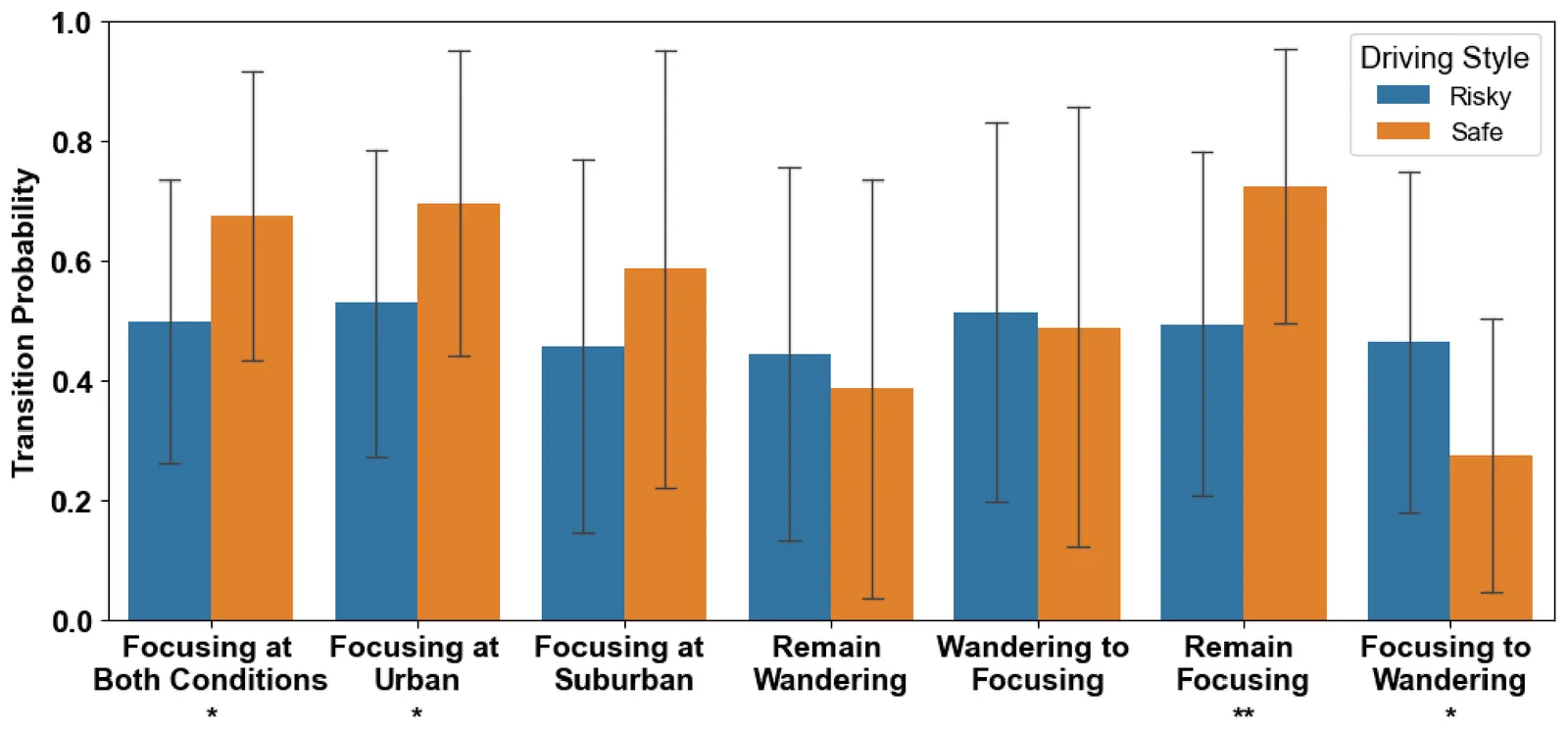
Probabilities of attentional states under different driving style. Bars represent means, and error bars represent standard error (* *p* < 0.05, ** *p* < 0.01).

Transition Probabilities. Each participant’s data contained a sequence of attentional state probes, and each sequence can generate four transition probabilities between two neighboring states: remain-wandering state, wandering switching to focusing state, remain-focusing state, and focusing switching to wandering state. Overall probabilities of mind focusing are shown in urban, suburban, and combined conditions in Figure 4.

A one-way ANOVA was performed for this comparison. We found that risky and safe drivers differed in their mind focusing probabilities in the combined urban and suburban conditions (Safe Median = 0.631, Risky Median = 0.511, *p* = 0.013) and in the urban condition (Safe Median = 0.714, Risky Median = 0.472, *p* = 0.028), but not in the suburban condition (Safe Median = 0.4, Risky Median = 0.5, *p* = 0.195). Overall, there was no significant difference (*p* = 0.550) in the propensity to remain in a mind-wandering state once in that state, and both groups showed similar tendencies to regain focus from a mind-wandering state (*p* = 0.796). However, the result revealed a significant difference between the groups in their ability to maintain focus (Safe Median = 0.772, Risky Median = 0.5, *p* = 0.003) and in the propensity to transition from a focusing state to a mind-wandering state (Safe Median = 0.227, Risky Median = 0.465, *p* = 0.015). Safe drivers remaining in the focusing state achieved the highest transition probability (77.3%) and the lowest probability (22.7%) for transitioning from focusing to the mind-wandering state.

### 3.3. Effects of Attentional States on Physiological and Driving Signals

Driving measures. Table 2 shows the driving measure results. The Speed Range was significantly lower in the mind wandering group (M = 4.499) than in the mind focusing group (M = 5.543, *p* = 0.023). The Speed STD was also significantly lower in the mind wandering group (M = 1.282) than in the mind focusing group (M = 1.544, *p* = 0.015).

Physiological Signals. Among six physiological sensors, three demonstrated significant differences. In terms of EDA phasic response, both the EDA Phasic Range and EDA Phasic STD were significantly lower in the mind wandering group than in the mind focusing group (Range: M = 0.045, CI = [0.026, 0.071], U = 51,073, z = −2.338, *p* = 0.019; STD: M = 0.013, CI = [0.008, 0.02], U = 50,516, z = −2.308, *p* = 0.021). The Pupil Diameter Median was significantly lower in the mind wandering group (M = 4.386, CI = [4.244, 4.487]) than in the mind focusing group (M = 4.602, CI = [4.511, 4.749], U = 54,644, z = −3.977, *p* < 0.001). However, all HRV measures (standard deviation of all normal to normal intervals (SDNN), root mean square of successive differences between normal heartbeats (RMSSD), standard deviation of successive differences between normal heartbeats (SDSD)) were significantly higher in the mind wandering group than in the mind focusing group (SDNN: M = 68.488, CI = [60.609, 79.904], U = 71,327, z = 2.195, *p* = 0.028; RMSSD: M = 76.534, CI = [58.623, 88.704], U = 71,282, z = 2.179, *p* = 0.029; SDSD: M = 77.043, CI = [59.556, 89.801], U = 71,276, z = 2.177, *p* = 0.03).

### 3.4. Effects of Driving Styles on Physiological Signals

Driving measures. Table 3 shows the driving measure results. The speed median was significantly higher in the risky group (Median = 21.62) than in the safe group (Median = 18.12, U = 58,503, z = −3.404, *p* < 0.001). The distance traveled was significantly higher in the risky group (Median = 127.65) than in the safe group (Median = 109.08, U = 58,236, z = −3.436, *p* = 0.001).

Physiological Signals. Various measures were compared between the two distinct driving styles. In terms of EDA, the Tonic Range, Tonic STD, Phasic Range, and Phasic STD were all significantly lower in the risky driving group than in the safe driving group (*p* < 0.001 for all). The same trend was observed for RR measures (RR Range, RR STD, RR Amplitude), where all measures were significantly higher in the risky group (*p* < 0.001 for all). For HRV measures, the SDNN, RMSSD, and SDSD were significantly higher in the risky driving group than in the safe driving group (*p* < 0.001 for all). The EMG Mean Absolute Value was significantly higher in the risky group (Median = 0.613, CI = [0.562, 0.749], U = 51,069, z = −4.155, *p* < 0.001) than in the safe group (Median = 0.542, CI = [0.469, 0.582]). For the eye-tracking measures, the Pupil Diameter Median was significantly higher in the risky group (Median = 4.649, CI = [4.541, 4.759], U = 54,458, z = −4.795, *p* < 0.001) than in the safe group (Median = 4.333, CI = [4.215, 4.483]). Both the mean and standard deviation of fixation duration were significantly higher in the risky group than in the safe group (mean: Median = 578.92, CI = [513.08, 656.12], U = 66,063, z = −2.209, *p* = 0.027; STD: Median = 484.83, CI = [450.51, 574.21], U = 56,383, z = −2.122, *p* = 0.034).

### 3.5. Classification of Attentional State and Driving Style

The classification analysis compares the sensor sets and classifier types under both attentional state labels and driving style labels. This analysis also performs a time window sensitivity analysis to select the optimal pre-probe time window.

**Figure 5:**
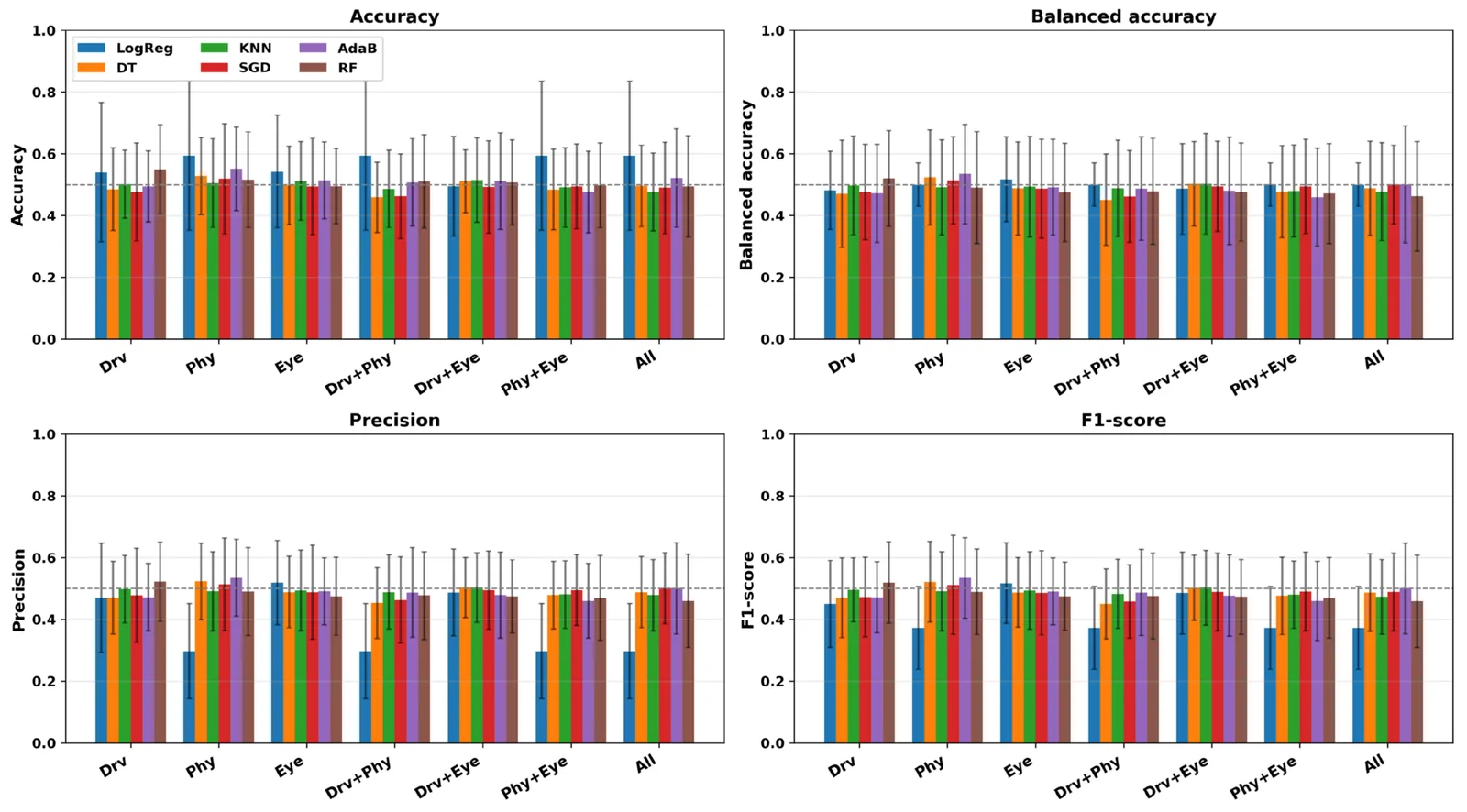
Classification performance for attentional states prediction under a 10 s pre-probe window. Metrics, including accuracy, balanced accuracy, macro precision, and macro F1-score, are reported across seven sensor sets and six classifiers, with the dashed line indicating 0.5 chance-level performance. Although the physiology-only sensor set showed the highest performance, overall performance remained weak.

Figure 5 summarizes the primary 10 s window results for attentional state classification. At this window length, most sensor-set/classifier combinations remained close to chance-level balanced accuracy. The best balanced accuracy was approximately 0.53, indicating that attentional state detection from short pre-probe windows remains difficult.

**Figure 6:**
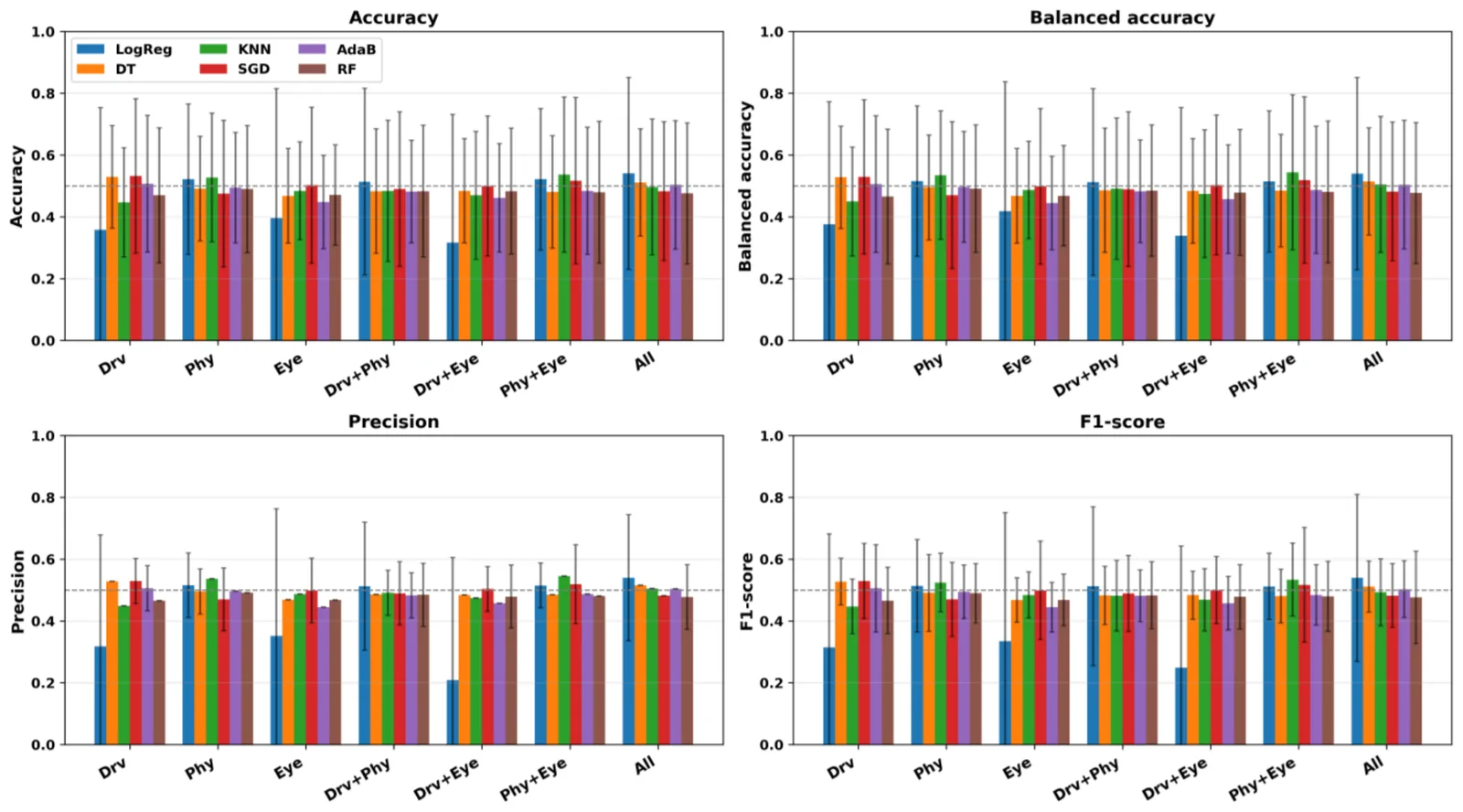
Classification performance for driving style prediction using 10 s pre-probe windows. Metrics, including accuracy, balanced accuracy, macro precision, and macro F1-score, are reported across seven sensor sets and six classifiers, with the dashed line indicating 0.5 chance-level performance. Physiological features showed the most consistent performance.

**Figure 7:**
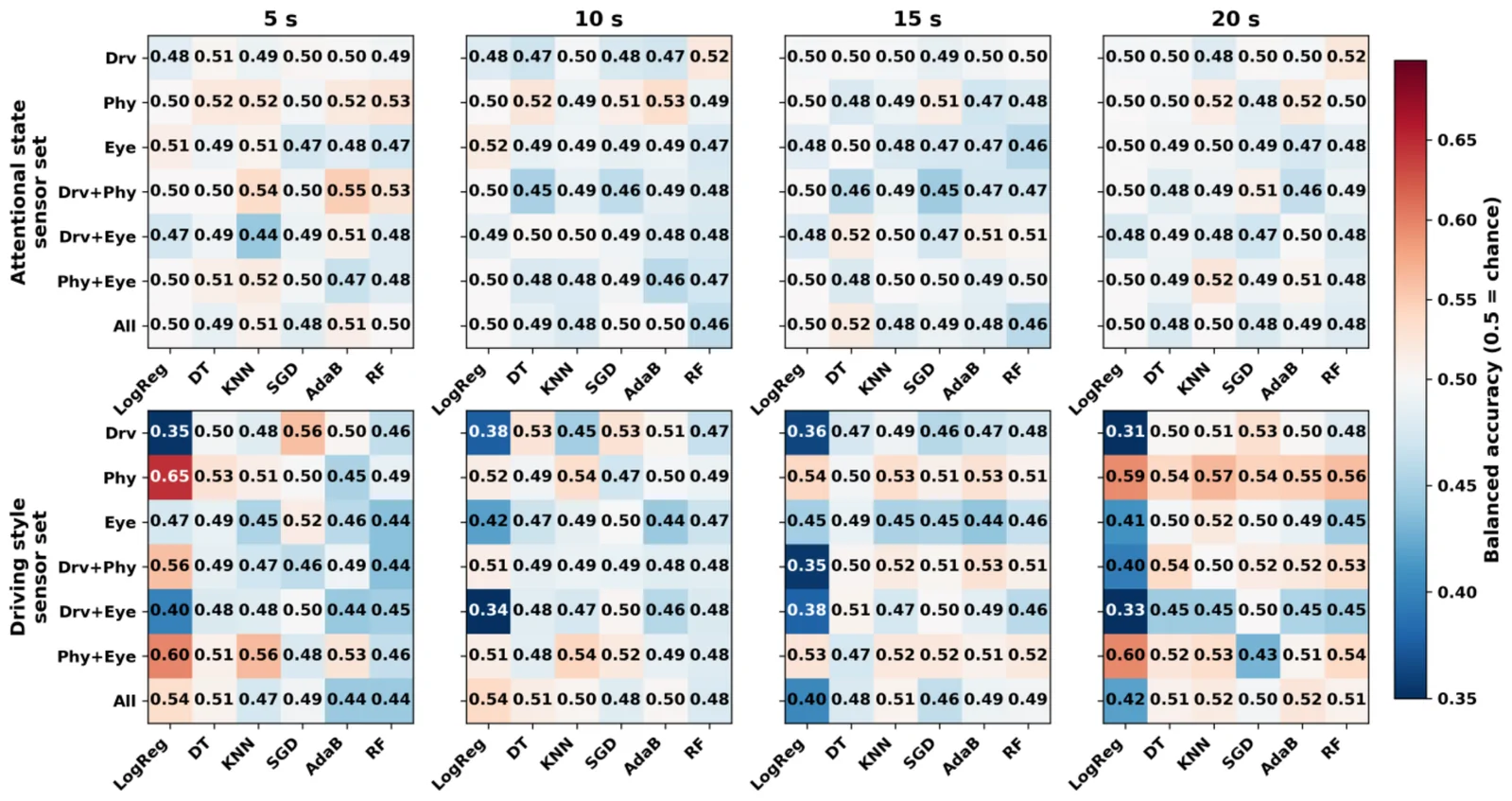
Balanced accuracy across sensor sets, classifiers, time windows, and prediction targets. The best attentional state result was obtained with driving + physiological features and AdaBoost at 5 s, whereas the best driving style result was obtained with physiology-only features and logistic regression at 5 s. LogReg: logistic regression; DT: decision tree; SGD: stochastic gradient descent; AdaB: Adaboost; RF: random forest.

Figure 6 reports the 10 s driving style classification metrics. At the primary window, most balanced-accuracy values clustered near chance, whereas the 5 s physiology-only logistic-regression model in Figure 7 provided the strongest overall driving style result. This pattern suggests that physiology carries the most consistent style-related information, but the effect is sensitive to temporal window and classifier choice.

Figure 7 presents the time window sensitivity analysis across 5, 10, 15, and 20 s windows. The best attentional state configuration was driving + physiological features with AdaBoost at the 5 s window (balanced accuracy = 0.55). Driving style classification showed clearer physiological sensitivity: the best configuration was physiology-only features with logistic regression at the 5 s window (balanced accuracy = 0.65). The results also show that longer windows did not uniformly improve performance.

**Figure 8:**
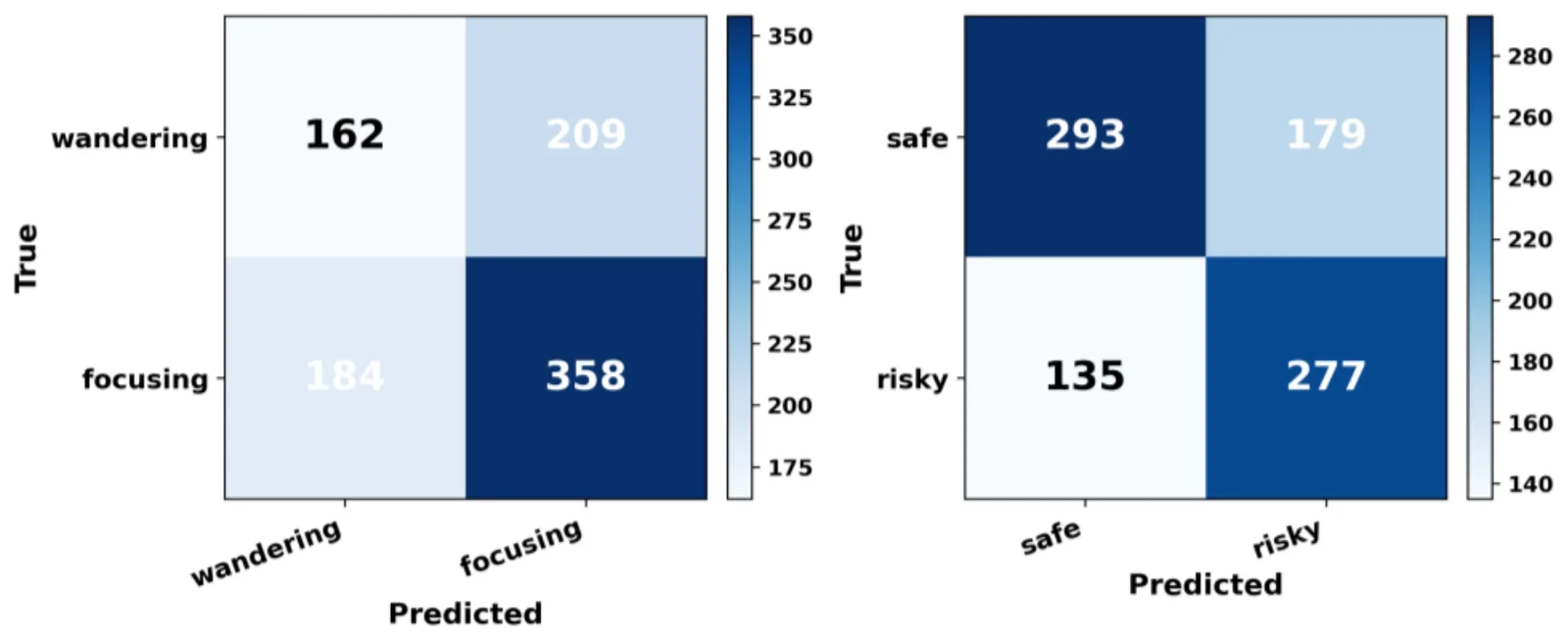
Confusion matrices for the best-performing configurations. The attentional state model achieved weak discrimination (balanced accuracy = 0.55), whereas the driving style model achieved modest discrimination (balanced accuracy = 0.65).

The confusion matrices of the best configurations are shown in Figure 8. For attentional state, the best model correctly classified 162 wandering windows and 358 focusing windows but also produced 209 wandering-to-focusing and 184 focusing-to-wandering errors, yielding a balanced accuracy of 0.55. For driving style, the best model correctly classified 293 safe-driver windows and 277 risky-driver windows, with 179 safe windows predicted as risky and 135 risky windows predicted as safe, yielding a balanced accuracy of 0.65.

**Figure 9:**
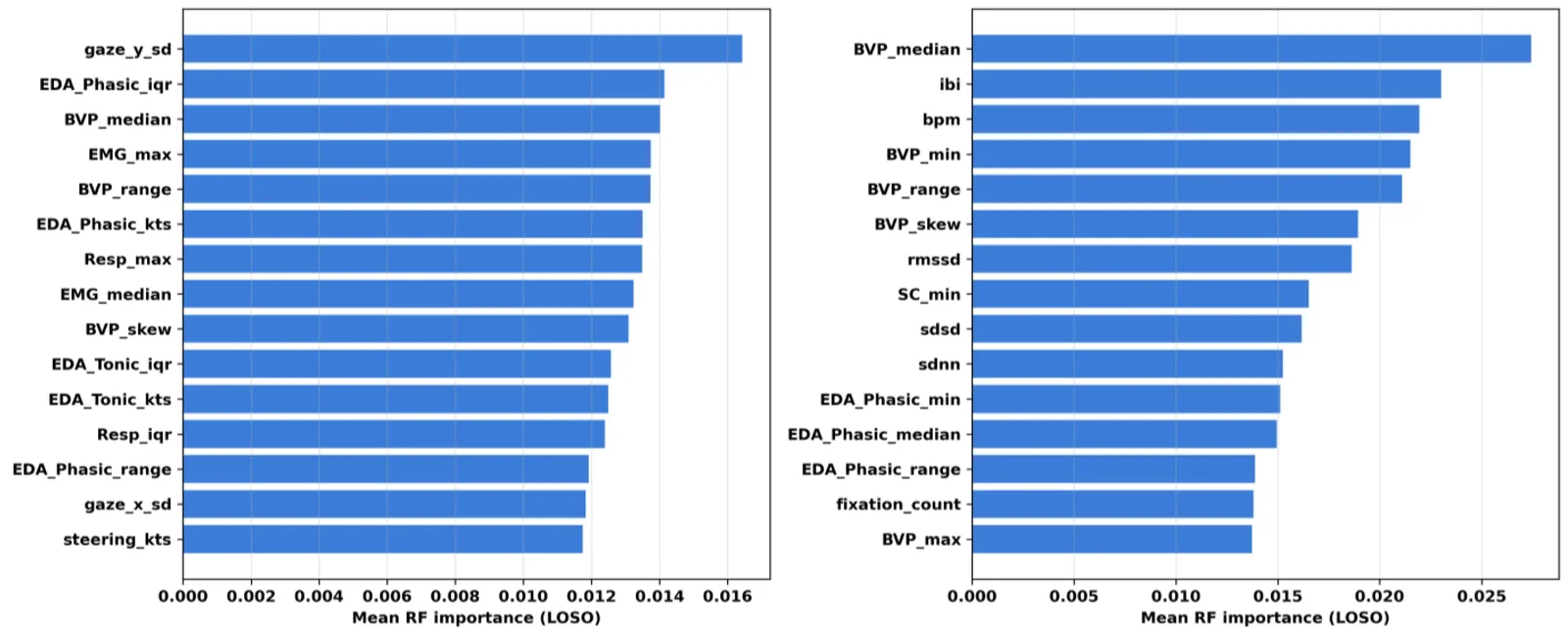
Feature importance based on the prediction of attentional state (**left**) and driving style (**right**). Attentional state prediction depended most on gaze variability and autonomic or muscular features, whereas driving style prediction depended most on cardiovascular and electrodermal features.

Random-forest feature importance is reported in Figure 9. For attentional state, the most influential features included vertical gaze variability, EDA phasic IQR, BVP median/range/skewness, EMG maximum/median, respiration maximum/IQR, and steering kurtosis. For driving style, the strongest features were BVP median, inter-beat interval, bpm, BVP minimum/range/skewness, RMSSD, SC minimum, SDSD, SDNN, EDA phasic features, fixation count, and BVP maximum. Attentional state inference depended on both gaze variability and autonomic/muscular responses, whereas driving style inference was dominated by cardiovascular and electrodermal features.

## 4. Discussion

This driving simulator study assessed how attentional state and driving style jointly manifest across multimodal sensor responses in a one-hour urban and suburban environment. The discussion is organized around the three main contributions of this study.

Table 4 summarizes the classification results referenced throughout this discussion. For each prediction target, attentional state and driving style, it reports the best-performing sensor configuration, the corresponding balanced accuracy, and a practical interpretation of that value, providing a compact reference point for the sensor-selection conclusions developed in the subsections below.

### 4.1. Style-Dependent Attention Dynamics

Our first analysis characterizes how attention dynamics differ by driving style, which can further indicate the driver profile for developing training or interventions. Recent reviews have noted that style-dependent attention dynamics receive limited attention in the driving literature, despite their relevance to in-vehicle monitoring [3,20].

Regarding the overall rate of mind wandering, our results showed that the rates in the risky and safe groups were 49% and 36%, respectively. We interpret this pattern in light of differences in driving task demand and environment. Probe-based mind-wandering studies reported this specifically under monotonous, low-traffic highway conditions designed to minimize task demand [26]. Our route is structured with urban and suburban conditions that required frequent speed changes, stop signs, and hazard monitoring.

This interpretation is consistent with task demand accounts of mind wandering. Higher perceptual and cognitive load causes less off-task thought and suppresses mind-wandering frequency. The high-density driving caused significantly less mind wandering than low-density driving [5]. In addition, the probe wording and probe frequency also differ across studies and contribute substantial measurement variance of their own, depending on the response options and probe instructions used [27].

Results from the driving parameters reveal that mind wandering is associated with a more conservative driving profile. This suggests that drivers maintain a subconscious compensatory mechanism to offset the reduced attention to driving. This momentary, within-state slowing is distinct from the trait-level pattern reported by [4], who found that a greater mind-wandering tendency predicts faster mean speed. However, other studies have identified driving measures, specifically lateral deviation metrics, as being more sensitive than physiological measures [28]. Factors such as curved roads in real cities can limit lane deviation data acquisition, and portable devices that cannot access vehicle-related measures make these parameters less reliable.

Notable differences were found between different attentional states via physiological signals, including EDA, pupil diameter, and HRV. Reduced EDA phasic responses and reduced pupil diameter during mind wandering suggest decreased cognitive engagement and workload. These findings are only partially consistent with prior physiological studies of mind wandering. For example, one study showed no significant difference in pupil size and mean heart rate when comparing mind wandering and attentive states [29], and this be partially due to methodological factors such as participant number, simulated driving duration, and the probe numbers, and the selection of driving events.

### 4.2. Driving Style as a Multimodal Trait

Our second analysis tests whether MDSI-defined driving style corresponds to objective multimodal features. The relationship between driving styles and physiological signals provides an intriguing perspective for research seeking to infer how individual driving behaviors correlate with specific physiological signals. Consistent with the result of our study, one prior study found that the summation of amplitudes of significant skin conductance responses was lower, but the number of significant skin conductance responses was higher in the risky group than in the safe group, indicating the mixed evidence for the nature of driving style. Recent studies have reported stable style profiles across cultures and professional driver populations, supporting the view that style is a relatively stable behavioral trait [30,31]. Our results extend this view by showing that the same MDSI labels are also recoverable from physiology alone at a balanced accuracy of 0.65.

The eye-tracking measures, such as larger pupil diameter, reflect the increased cognitive demands of risky drivers. There are no significant differences in the frequency and duration of glances to roadway information based on the mental state of the individual or the task relevancy of the information [32], which is consistent with our findings. This observation is also consistent with reports that gaze metrics carry style-related variance distinct from attentional state [33].

### 4.3. Task- and Subgroup-Specific Sensor Configurations

Our third analysis evaluates how sensor combinations perform across two prediction targets and across driver subgroups. From Figure 5 and Figure 6, it is evident that the combination of physiological and eye-tracking features is the most effective in predicting attentional state across both safe and risky groups. This interconnectedness of physiological responses and eye movements in reflecting attention levels while driving aligns with other literature on distracted driving detection [34]. However, we observed the performance disparity in driving features, where the classification accuracy dropped substantially from the safe group to the risky group, suggesting that driving alone might not be universally reliable across all groups, given the natural individual difference in the driver (safe vs. risky). This pattern is consistent with recent EEG-based driver distraction reviews that note the lack of a universally optimal feature set across populations [35].

The feature-importance analysis refines the interpretation of sensor usefulness. Attention-related models relied on gaze variability together with EDA, BVP, EMG, respiration, and steering features, consistent with the view that short-term attentional state is reflected in both visual allocation and autonomic/motor regulation. Driving style models were dominated by BVP and HRV features, with additional contributions from EDA and fixation count. This pattern suggests that physiology may capture stable style-related differences more consistently than vehicle-control features alone, while eye tracking contributes more to momentary attentional state characterization.

This is consistent with other literature classifying driving styles via physiological signals. Liang and Lin achieved 79.8% accuracy in classifying driving styles via physiological and EEG signals using a linear discriminant analysis algorithm [10]. Recent work on aberrant driving prediction using HRV further supports the central role of physiological features for style-related inference [36].

### 4.4. Limitation, Future Work, and Practical Implications

The appropriate time window representing a stable attentional state remains a topic of debate in the literature. Our probes were spaced 120–180 s apart, which is shorter than the average mind-wandering episode of roughly 10 min in some experience-sampling studies. This study reported that the neural precursors of mind wandering have been reported to emerge 4–10 min before a subjective report [23]. Therefore, it is possible that our probing interval curtailed some naturally occurring mind-wandering episodes before they fully developed or stabilized. However, other probe-based mind-wandering studies used comparably short inter-probe intervals and did not find the evidence that probing frequency artificially suppresses mind wandering, including durations ranging from 2.5 to 10 s [24], and between 15 and 45 s [37]. Our interval was chosen to balance sampling density against the intrusiveness of probing on driving performance. Future studies could nonetheless use variable or longer inter-probe intervals, or continuous/self-caught reporting, to further rule out this possibility.

We acknowledge that the binary driving label approach only shows modest-to-moderate correspondence with observed on-road driving behavior in other validation studies [38]. Therefore, the ‘safe’ and ‘risky’ labels should be understood as proxies for a behavioral tendency. Mean-split dichotomization further discards within-group variation and is sensitive to the specific sample’s score distribution.

From a deployment perspective, the performance itself needs to be interpreted narrowly. This study achieved a balanced accuracy of 0.55 for attentional state classification and 0.65 for driving style classification. These values are broadly consistent with the literature, which indicates that detecting cognitive driver distraction is more difficult than detecting overt, behavior-based distraction. Overt distraction involving clearly visible head-pose changes is often reported at above 90–100% accuracy [39]; gaze-based systems that infer distraction from dashboard-fixation variance report 68–81% accuracy [40]; and eye-tracking-only configurations without vehicle context report accuracies in the mid-60% to 70% range [41]. Across these studies, accuracy improves consistently when facial or physiological cues are fused with vehicle- or ADAS-derived context. Accordingly, the present results can be interpreted as follows: when vehicle-status and driving-environment context is limited or unavailable, physiology-only wearable devices remain comparatively unreliable [42]. This finding further suggests that when vehicle-status and driving-environment context is limited or unavailable, wearable-only driver-state estimation may remain unreliable. Future driver-state monitoring systems should therefore embed wearable physiological signals within broader ADAS or vehicle-context frameworks [43].

## 5. Conclusions

This study examined how driving style and attentional state jointly shape multimodal sensor responses during prolonged simulated driving. Three findings should be emphasized. First, MDSI-defined driving style corresponded to objective multimodal signatures, with physiology-only features producing the strongest driving style classification result (balanced accuracy = 0.65). Second, attention dynamics differed by driving style, with risky drivers showing a greater tendency to drift from focusing to mind wandering. Third, sensor usefulness depended on the prediction target and driver subgroup: attentional state classification remained weak (balanced accuracy = 0.55), whereas driving style classification showed modest physiological sensitivity. These results do not support standalone real-time detection using wearable sensors alone. Instead, they provide empirical guidance for sensor selection and suggest that wearable driver-state estimation should be integrated with vehicle-control, facial, gaze, and ADAS-derived contextual information. Future work should extend the protocol to naturalistic driving, test longer and variable probe intervals, and develop temporal models of attention transitions over longer periods.

## Figures and Tables

**Table 1:** Features generated from driving, physiological, and eye-tracking signals.

Category	Measure (Unit)	Definition
Driving	Speed (m/s)	The distance covered per unit of time
	Accelerator (0–1)	The amount of pressure applied to the accelerator pedal
	Distance Traveled (m)	Total distance covered during a period
Temperature	Skin Temperature (°C)	The surface temperature of the skin, which can reflect physiological arousal and emotional state
BVP	SDNN (ms)	Standard deviation of all NN (normal to normal) intervals, representing overall heart rate variability
	RMSSD (ms)	Root mean square of successive differences between normal heartbeats, representing short-term variability
	SDSD (ms)	Standard deviation of successive differences between normal heartbeats, another measure of short-term variability
EDA	Tonic (µS)	The stable, slow-changing aspect of electrodermal activity, representing overall arousal level
	Range (µS)	The difference between the highest and lowest electrodermal response in a given period
Resp	Amplitude	The difference between the peak (maximum inspiration) and trough (maximum expiration) in a respiratory cycle
Eye	Pupil Diameter (mm)	The size of the pupil, which can reflect emotional state and cognitive load
	Fixation Duration (ms)	The length of time for which gaze remains fixed on a unique location
EMG	Mean Absolute Value (millivolts)	The average rectified value of the electromyogram signal, often used to quantify muscle activity

**Table 2:** Significant differences in driving, physiological, and eye-tracking measures between attentional states.

Feature Name	Group	Median [95%CI]	U	Z	*p*
Speed Range	Wandering	4.499 [3.949, 5.144]	58,815	−2.277	0.023
	Focusing	5.543 [4.863, 6.036]			
Speed STD	Wandering	1.282 [1.158, 1.466]	57,881	−2.44	0.015
	Focusing	1.544 [1.378, 1.772]			
EDA Phasic Range	Wandering	0.045 [0.026, 0.071]	51,073	−2.338	0.019
	Focusing	0.084 [0.059, 0.121]			
EDA Phasic STD	Wandering	0.013 [0.008, 0.02]	50,516	−2.308	0.021
	Focusing	0.025 [0.018, 0.033]			
Pupil Diameter	Wandering	4.386 [4.244, 4.487]	54,644	−3.977	<0.001
	Focusing	4.602 [4.511, 4.749]			
SDNN	Wandering	68.488 [60.609, 79.904]	71,327	2.195	0.028
	Focusing	62.075 [57.171, 67.483]			
RMSSD	Wandering	76.534 [58.623, 88.704]	71,282	2.179	0.029
	Focusing	61.818 [54.904, 69.813]			
SDSD	Wandering	77.043 [59.556, 89.801]	71,276	2.177	0.03
	Focusing	62.291 [55.294, 70.211]			

**Table 3:** Significant differences in driving, physiological, and eye-tracking measures between safe and risky driver groups.

Feature Name	Group	Median [95%CI]	U	Z	*p*
Speed median	Safe	18.12 [16.68,19.80]	58,503	−3.404	<0.001
	Risky	21.62 [20.32,23.14]			
Distance Traveled	Safe	109.08 [99.32,118.03]	58,236	−3.436	<0.001
	Risky	127.65 [118.84,137.77]			
EDA Tonic Range	Safe	0.17 [0.151, 0.198]	81,388	7.008	<0.001
	Risky	0.065 [0.045, 0.079]			
EDA Tonic STD	Safe	0.05 [0.043, 0.058]	80,919	6.912	<0.001
	Risky	0.018 [0.014, 0.023]			
EDA Phasic Range	Safe	0.118 [0.079, 0.162]	70,933	4.447	<0.001
	Risky	0.038 [0.024, 0.054]			
EDA Phasic STD	Safe	0.034 [0.024, 0.043]	69,878	4.341	<0.001
	Risky	0.011 [0.007, 0.016]			
RR Range	Safe	2.355 [2.185, 2.474]	55,544	−3.905	<0.001
	Risky	2.824 [2.631, 2.952]			
RR STD	Safe	0.685 [0.655, 0.716]	54,112	−4.304	<0.001
	Risky	0.819 [0.777, 0.874]			
RR Amplitude	Safe	2.355 [2.221, 2.476]	55,544	−3.905	<0.001
	Risky	2.824 [2.631, 2.954]			
SDNN	Safe	51.667 [48.645, 57.915]	46,349	−7.343	<0.001
	Risky	86.71 [74.643, 99.336]			
RMSSD	Safe	47.058 [42.79, 53.917]	45,044	−7.796	<0.001
	Risky	94.947 [81.112, 106.74]			
SDSD	Safe	47.298 [43.055, 53.745]	45,045	−7.796	<0.001
	Risky	95.474 [81.722, 107.39]			
EMG Mean Absolute Value	Safe	0.542 [0.469, 0.582]	51,069	−4.155	<0.001
	Risky	0.613 [0.562, 0.749]			
Pupil Diameter Median	Safe	4.333 [4.215, 4.483]	54,458	−4.795	<0.001
	Risky	4.649 [4.541, 4.759]			
Fixation Duration Mean	Safe	534.36 [481.46, 567.09]	66,063	−2.209	0.027
	Risky	578.92 [513.08, 656.12]			
Fixation Duration STD	Safe	415.83 [367.69, 464.11]	56,383	−2.122	0.034
	Risky	484.83 [450.51, 574.21]			

**Table 4:** Summary of the best-performing sensor configurations and practical interpretation.

Prediction Target	Best Configuration	Balanced Accuracy	Practical Interpretation
Attentional state	Driving + physiological features; AdaBoost; 5 s window	0.55	Weak discrimination; not sufficient for standalone real-time intervention
Driving style	Physiological features; logistic regression; 5 s window	0.65	Modest between-subject style signal; useful for sensor-selection interpretation

## Data Availability

Datasets in the study are available from the corresponding author upon reasonable request. The feature-extraction and classification code will be made available in a public GitHub repository (https://github.com/Luke-zhuwc/Mind-Wandering-Classification-in-Driving-Simulations (accessed on 9 September 2026)).
